# Supplementary material for: Critical role of smoking and household dampness during childhood for adult phlegm and cough: a research example from a prospective cohort study in Great Britain
Source: BMJ Open. 2014 Apr 19;4(4):e004807. doi: 10.1136/bmjopen-2014-004807 (PMC4010851; doi:10.1136/bmjopen-2014-004807)
Supplement: Author's manuscript [file bmjopen-2014-004807.draft_revisions.pdf]

Critical roles of smoking and household dampness during childhood on adult respiratory health: A research example from the 1970 British Birth Cohort Study

Journal:	*BMJ Open*
Manuscript ID:	bmjopen-2014-004807
Article Type:	Research
Date Submitted by the Author:	06-Jan-2014

Complete List of Authors:	Cable, Noriko; UCL, Epidemiology and Public Health<br>Bartley, Mel; UCL, Epidemiology and Public Health<br>Kelly, Yvonne; UCL, Epidemiology and Public Health<br>Sato, Yuki; National Institute for Environmental Studies, Centre for Environmental Health Sciences<br>Sacker, Amanda; UCL, Epidemiology and Public Health

**Primary Subject Heading**:	Epidemiology
Secondary Subject Heading:	Respiratory medicine
Keywords:	BCS70, household dampness, adult respiratory health, Life course study

SCHOLARONE™ Manuscripts

For peer review only

Critical roles of smoking and household dampness during childhood on adult respiratory health: A research example from the 1970 British Birth Cohort Study

Authors: Noriko Cable[1], Mel Bartley[1], Yvonne Kelly[1], Yuki Sato[2], Amanda Sacker[1]

[1]Research Department of Epidemiology and Public Health, University College London 1-19 Torrington Place, London WC1E 6BT United Kingdom: Noriko Cable PhD, Senior Research Fellow, Yvonne Kelly PhD, Professor of Life course Epidemiology, Mel Bartley PhD, Professor of Medical Sociology, Amanda Sacker PhD, Professor of Quantitative Social Science

[2]Centre for Environmental Health Sciences, National Institute for Environmental Studies, 16-2 Onogawa, Tsukuba-city, Ibaragi 305-8506, Japan: Yuki Sato PhD, Researcher

Correspondence to: n.cable@ucl.ac.uk

For peer review only

Abstract

Objective: To examine independent associations between childhood exposures to smoking and housing dampness and adult respiratory health.

Design: A prospective cohort study.

Participants: 7,320 of the British cohort who were born during one week in 1970 and had complete data for childhood and adult information.

Primary outcome measures: Experiences of phlegm and coughing over the previous three months were assessed using the questions from the MRC questionnaire when the cohort participants were 29 years of age. Four response patterns (no symptoms, phlegm only, cough only, both symptoms present) were created based on the responses to these questions.

Results: Childhood smoking and exposure to household dampness at age 10 were associated with phlegm-related symptoms, while exposure to adult smokers in the household was associated with cough-related symptoms. These associations were independent from adult smoking, childhood respiratory health, early social background, and sex. Although current smoking at age 29 was the largest contributor to the outcome, overall effects from childhood exposures, notably household dampness (χ^2=28.79, DF=2, p<0.001) were significant for co-occurring phlegm and cough.

Conclusions: Our findings give support to current public health interventions for adult smoking and raise concerns about the long-term effects of a damp home environment on the respiratory health of children.

For peer review only

Strengths and limitations of the study

- This study uses a large sample with the prospective cohort design, allowing to examine the longitudinal associations between childhood exposures (parental and childhood smoking, household dampness) and adult respiratory health without introducing recall bias.
- Confounders (Sex, childhood SES, respiratory difficulties at birth, childhood respiratory health) and adult smoking were accounted to identify significant association between exposures at age 10(childhood smoking, parental smoking, and household dampness) and adult respiratory health at age 29.
- The outcome measure to indicate adult respiratory health is based on two respiratory symptoms (phlegm and cough) from a well-established questionnaire and not indicative of a particular respiratory disease or lung function.

For peer review only

Introduction

Smoking cessation has been supported as the main preventive measure to reduce smoking-related illnesses and mortality[1]. Previous findings have suggested additional risk factors for adult respiratory health. There are longitudinal links between exposure to parental smoking[2-5] or social disadvantage[6-8] during childhood and adult respiratory health, while research evidence to support the association between indoor dampness and respiratory health is mainly cross-sectional[9-14].

Examining the life course respiratory health of the birth cohort of Britons born in 1946, Mann, Wadsworth and Colley[7] reported that the greatest contribution to adult respiratory health was from proximal risk factors such as adult smoking rather than from distant risk factors such as child respiratory health or social disadvantages. Since adult respiratory health is largely determined by child respiratory health[15], additional longitudinal effects of childhood exposures, such as smoking and exposure to household damp on adult respiratory health may be small.

However, not many studies have used repeated measures when childhood respiratory health was taken into account. The contribution of childhood exposures to adult respiratory health can be better estimated if a similar respiratory measure is used to assess both child and adult respiratory health. Here we use respiratory symptoms of phlegm and cough occurring in childhood and in adulthood to examine the degree of contribution to adult respiratory health from exposure to smoking and household damp during childhood. We used a British cohort who were born in 1970 and examined two hypotheses.

1. Household dampness, exposure to adult cigarette smoke during childhood, and childhood smoking will have long-term associations with adult respiratory health over and above childhood respiratory health and smoking in adulthood.

For peer review only

2. Overall effects from those childhood factors are larger than the effects of smoking in adulthood.

Method:

Study design, setting and participants

We used information obtained from a prospective cohort study, the 1970 British Cohort Study (BCS70). Detailed information about research design and collected data is given by Elliot and Shepherd[16]. BCS70 targets British residents who were born during one week in 1970; data have been collected regularly across their life course. We used datasets collected at birth[17], at age 10[18] and at age 29[19] to extract study variables. In this study, 7,320 available cases which contain all of the study information were used for the analyses.

Ethical approvals of the study

Data collection at birth and at age 10 was reviewed internally, while ethical approval from the London Multi-centre Research Ethics Committee was obtained for the age 29 survey[20].

Explanatory factors

We used three childhood exposures (child's own smoking, number of adult smokers in the household, household dampness) as explanatory factors. Information about these factors was collected when the cohort children were 10 years of age. A cohort child's own smoking status was indicated by the response to the question: 'Have you tried a cigarette?' Children who had tried a cigarette were coded as 1 as opposed to those who had not yet experimented with smoking (=0). The number of adult smokers in the household was derived through the response about the smoking status of the parents and other adults in the household obtained through a parental interview. This response was tabulated into three categories: 0 for no adult smokers, 1 for one adult, and 2 for two or more adults. In addition, the degree of household dampness was addressed via this parental interview, asking them to rate household dampness

For peer review only

as: none, slight, moderate, or marked. Only a few people responded that their house was 'moderately' damp; therefore, this response was included in the category of 'slight' dampness.

Adulthood factor

Information about adult smoking was collected when the cohort participants were 29 years of age. Their smoking status was indicated as 0 for current non-smokers and 1 for current smokers.

Outcome variable

Adult respiratory health, patterns of co-occurrence in phlegm and coughs, was the outcome of this study. Cohort participants were asked to respond whether they brought up phlegm or experienced coughs in the morning, during the day or at night in the winter when they were 29 years old. These respiratory questions were from the Medical Research Council Questionnaire and have been widely used, including in the previous cohort study (i.e. National Child Developmental Study). [21] We adapted g the method used by Strachan et al.[22] and created a variable to indicate presence of each respiratory symptom (=phlegm or cough), which cohort participants might have experienced at any time of day. After that we derived a variable with four response patterns of adult respiratory symptoms by combining the response to each variable: 0 being no respiratory symptoms, 1 being phlegm only, 2 being cough only, and 3 being both phlegm and cough, at age 29.

Confounders

We controlled for childhood respiratory health, sex, paternal social position. Previous respiratory health was indicated by presence of phlegm (0=no, 1=yes) or cough (0=no, 1=yes) when the cohort children were 10 years old. These variables were created through the responses (no, yes but for less than 3 months a year, yes for 3 months or more a year, not known) to the questions: 'Does the study child usually cough/bring up phlegm first thing in the morning/during the day or at night?' These questions are similar to ones from the MRC

For peer review only

questionnaire, with the omission of the phrase ‘in the winter’. A positive response to a particular respiratory question was treated as presence of the particular respiratory symptom (phlegm or cough), while the response ‘no’ was treated as absence of that respiratory symptom. The response ‘not known’ was regarded as missing and excluded from the analyses. We also included the presence of respiratory difficulties, including respiratory distress syndrome, at the birth of the cohort children (0=absent, 1=present) in the model.

Parental social position was indicated by paternal occupational position at the birth of the cohort child. We adapted the approach by Power et al.[23], classifying the Registrar General’s Social Classes of the cohort children’s father as: 1 – professional or managerial (I& II); 2 – skilled non-manual (III non-manual); 3 – skilled manual (III manual); 4 – partly skilled or unskilled (IV&V) and children who were born in a mother-only household. Midwives provided the information relevant to the birth of the cohort children, while the presence of cough or phlegm was assessed via the parental interview.

Statistical analysis

Multinomial logistic regression was used to identify associations between explanatory factors and patterns of adult respiratory symptoms (phlegm, cough, or both). We took those who did not have any respiratory symptoms as a reference category. A post-hoc estimate (Wald test) was performed to assess the overall effects of childhood and adult exposures on adult respiratory health. In our study men and women were analysed together, controlling for the effect of sex. All statistical analyses were performed using the Stata SE v12[24].

Results

Study characteristics

Approximately 2.5% of mothers of the cohort children reported that their children brought up phlegm at age 10, while it was 11% for cough (N=7320). It was 8.7% of the participants who reported co-occurring symptoms of phlegm and cough at age 29, while it was 2.9% for

For peer review only

phlegm only and 9.2% for cough. Assessing respiratory symptoms across the life course, there were small proportions of the participants who continuously experienced respiratory symptoms at both times (Table 1).

According to the maternal report less than half of the cohort children grew up in a smoke-free domestic environment, while household dampness of any degree was observed in around 17% of the houses of the cohort children. Many of the cohort participants did not smoke at 10 years of age, but around half of the cohort participants were current smokers at age 29. Demographically 48% of the participants were men and 30% of the participants had fathers with non-manual occupations. Many of the cohort participants did not experience respiratory difficulties when they were born.

Long-term associations between childhood factors and adult respiratory health

The results of multinomial logistic regression showed childhood factors were independently and specifically associated with adult respiratory health. (Table 2) The relative risk ratio (RRR) for childhood smoking was 1.45 (95%CI=1.02-2.05) for phlegm only and was 1.35 (95%CI=1.08-1.67) for co-occurring phlegm and cough. Growing up in a markedly damp house showed a similar, but stronger association with phlegm only (RRR=2.05, 95%CI=1.07-3.91) and co-occurring phlegm and cough (RRR=2.73, 95%CI=1.88-3.99).

Having adult smokers in the house when the cohort child was 10 years old was positively associated with all patterns of respiratory symptoms; however, having two or more adult smokers were significantly associated with adult cohort participants' respiratory symptoms that were cough only (RRR=1.28, 95%CI=1.04-1.58) or co-occurrence of phlegm and cough (RRR=1.32, 95%CI=1.06-1.64). These associations are independent of adult smoking and confounders. Adult smoking at age 29 was significantly associated with all of the respiratory response patterns.

For peer review only

Assessing the magnitude of childhood factors and current adult smoking on adult respiratory health

The results of Wald tests showed a significant contribution to co-occurring phlegm and cough at age 29 from the **overall effects** of childhood exposures, notably from household dampness at age 10 (χ^2=28.79, DF=2, p<0.001). (Table 3) Numbers of adult smokers in the household was the only childhood exposure that showed a significant contribution to all patterns of adult respiratory health at age 29; this was, however, marginal. Current smoking at age 29 had the largest impact on all types of adult respiratory symptoms (Phlegm only: χ^2=28.60, DF=1, p<0.001; Cough only: χ^2=96.49, DF=1 p<0.001; Co-occurrence of phlegm and cough: χ^2=171.66, DF=1, p<0.001) over and above childhood exposures, childhood respiratory health, sex, and other confounders.

Discussion

Key results

Using a British cohort born in 1970, we showed significant longitudinal associations between childhood exposures (child's own smoking, two or more adult smokers in the house, and marked household dampness) and patterns of adult respiratory health. These were independent of childhood respiratory health, adult smoking status, sex and social position of origin. Although the contribution from childhood exposures to adult respiratory health was relatively small compared to adult smoking, household dampness at age 10 showed a substantial contribution to co-occurring phlegm and cough at age 29.

Strengths and limitations

This study benefits from a substantially large sample size with a prospective design which limits recall bias. Questions from the MRC questionnaire were used to identify the patterns of respiratory symptoms (either phlegm or cough, or both symptoms present) at age 29, rather

For peer review only

than the presence of respiratory diseases. We consider that identifying respiratory symptoms of phlegm or cough at age 10, using similar questions to the adult questionnaire, enabled us to estimate associations between childhood exposures and adult respiratory health with considerable accuracy. The omission of the words 'in the winter' in the child version of the respiratory questions, however, may have over-identified the childhood cases of phlegm or cough. In addition, our findings relating to childhood smoking status should be interpreted carefully as they were obtained via self-report. An objective measure of lung function is not available in any sweeps of the BCS70 data which is also a limitation to our study.

Possible explanations and comparison with previous studies

We found that experimenting with smoking and exposures to marked household dampness at 10 years of age were longitudinally associated with phlegm-related symptoms at age 29, while having two or more adult smokers in the household showed detrimental associations with cough-related symptoms at the same age. These longitudinal associations are independent of previous respiratory health, adult smoking, and other confounders. It is worth noting that nearly 80% of the cohort participants (n=11648) did not have any respiratory symptoms at age 10 to start with, yet 20% of these participants had developed respiratory symptoms, phlegm or cough by age 29. This is somewhat similar to the findings by Guerra et al.[3] that lung function became markedly poorer at age 26 only when participants were exposed to both parental smoking at their birth and were smoking. It is possible that declines in respiratory health may not coincide with earlier exposures to smoking or household dampness. Our findings also add to the existing findings to link parental smoking and respiratory symptoms in adulthood [2 4] and indicate that the mode (active vs. second-hand) of childhood exposure to smoking may affect respiratory systems differently.

For peer review only

In addition, our finding linking household dampness with presence of phlegm in adulthood is similar to the findings by Sahlberg et al.[25] that adults exposed to household dampness showed increased occurrence of mucosal symptoms in 10 years. Information about the duration of exposure to indoor dampness is not available; however, it is likely that exposure to severe indoor dampness may have irritated the respiratory system of the cohort children, even though they were asymptomatic at that time.

Similarly to the study by Mann et al.[7], our finding supports longitudinal associations between childhood exposures and adult respiratory health; however, our study is the first to show a substantial contribution of childhood exposure to household dampness to co-occurring phlegm and cough at age 29.

Implications and conclusions

In summary, we support the longitudinal associations between childhood exposures to smoking and household dampness, and adult respiratory health. Although a large contribution to adult respiratory health was found from adult smoking, exposure to household dampness during childhood showed a substantial contribution to co-occurring phlegm and cough. Our findings support current public health interventions to reduce adult smoking, but also indicate that the management of childhood risk factors such as exposure to smoke (active or second-hand) and household dampness can be a way to prevent adults experiencing poor respiratory health.

For peer review only

Footnotes

Contributors: NC planned the study, analysed the data, and paricipated in writing and discussion. AS advised statistical analyses and participated in writing and discussion. MB, YK and YS participated in writing and discussion.

Funding information: This work was funded through the Economic and Social Research Council's International Centre for Life Course Studies in Society and Health (ES/J019119/1).

Conflicts of Interests: None declared

Ethical approval: BCS70 data are in public domain and accessible via the UK data archive. The data do not contain identifiable information and academic use of the BCS70 data does not require ethical approvals.

Data sharing statement: BCS70 data used in this study were managed by the Centre for Longitudinal Study and accessible via the UK data archive. The statistical code for derived variables is available from the correspondance author.

For peer review only

Table 1. Descriptive analyses (%) of explanatory and confounding variables by respiratory conditions (no symptoms, phlegm only, cough only, phlegm and cough) at age 29 (N=7,320)

	No symptoms % (n=5792)	Phlegm % (n=214)	Cough % (n=675)	Phlegm + Cough % (n=639)
**Explanatory variables**				
Smoking status at age 10				
Never	88.09	78.97	84.74	79.50
Smoked	11.92	21.12	15.26	20.50
Household dampness at age 10				
No dampness	84.34	83.18	84.15	76.37
Slight to moderate	13.33	11.68	12.40	16.91
Marked	2.33	5.14	3.41	6.73
Numbers of adult smokers in the household at age 10				
Zero	36.08	28.50	29.93	26.60
One	36.31	38.32	35.83	38.50
Two or more	27.61	33.18	34.22	34.90
Smoking status at age 29				
Non-smoker	50.36	29.44	29.19	19.56
Current smoker	49.64	70.56	70.81	80.44
**Confounders**				
Phlegm at age 10				
Absent	97.95	94.39	96.00	95.62
present	2.05	5.61	4.00	4.38
Cough at age 10				
Absent	90.06	85.51	85.63	83.72
Present	9.94	14.49	14.37	16.28
Respiratory difficulties at birth				
Absent	97.86	97.66	97.78	97.81
Present	2.14	2.34	2.22	2.19
Social position of origin				
I & II	18.42	20.56	14.67	13.77
III non-manual	14.50	11.21	13.04	10.33
III manual	45.51	42.99	46.96	48.36
IV&V + no male head	21.56	25.23	25.33	27.54
Sex				
Male	45.98	67.76	45.63	63.85
Female	54.02	32.24	54.37	36.15

For peer review only

Table 2. Associations between child and adult risk factors and adult respiratory conditions expressed by relative risk ratios (RRR) and 95%CI obtained by multinomial logistic regression (N=7320)

	No symptoms (n=5792)	Phlegm only (n=214)		Cough only (n=675)		Phlegm + Cough (n=639)	
		Bivariate	Fully adjusted	Bivariate	Fully adjusted	Bivariate	Fully adjusted
**Explanatory factors**							
Smoking status at age 10 (Reference: Not smoked)	Ref	1.97 (1.41-2.76)	1.45 (1.02-2.05)	1.33 (1.01-1.67)	1.12 (0.89-1.41)	1.91 (1.55-2.35)	1.35 (1.08-1.67)
Household dampness at age 10 (Reference: no dampness)							
Slight to moderate	Ref	0.89 (0.58-1.36)	0.82 (0.54-1.27)	0.94 (0.74-1.19)	0.85 (0.67-1.09)	1.40 (1.12-1.75)	1.24 (0.99-1.56)
Marked	Ref	2.24 (1.19-4.21)	2.05 (1.07-3.91)	1.47 (0.93-2.30)	1.26 (0.80-1.99)	3.19 (2.23-4.55)	2.73 (1.88-3.99)
Numbers of adult smokers in the household at age 10 (reference: Zero)							
One	Ref	1.34 (0.6-1.87)	1.23 (0.88-1.74)	1.19 (0.98-1.45)	1.07 (0.87-1.30)	1.44 (1.17-1.76)	1.20 (0.97-1.49)
Two or more	Ref	1.52 (1.07-2.16)	1.36 (0.95-1.95)	1.49 (1.22-1.83)	1.28 (1.04-1.58)	1.71 (1.39-2.12)	1.32 (1.06-1.64)
Current smoker at age 29 (Reference: Non-smoking)	Ref	2.43 (1.80-3.28)	2.29 (1.69-3.09)	2.46 (2.07-2.93)	2.41 (2.02-2.88)	4.17 (3.41-5.11)	3.95 (3.22-4.85)
**Confounders**							
Phlegm present at age 10 (Reference: Absent)	Ref	--	2.62 (1.28-5.39)	--	1.60 (0.99-2.58)	--	1.63 (1.01-2.64)
Cough present at age 10 (Reference: Absent)	Ref	--	1.14 (0.72-1.81)	--	1.34 (1.03-1.74)	--	1.45 (1.12-1.89)
Sex (Reference: Men)	Ref	--	0.42 (0.31-0.56)	--	1.03 (0.87-1.21)	--	0.49 (0.41-0.59)
Respiratory difficulties at birth	Ref	--	1.00	--	1.06	--	0.97

For peer review only

(Reference: Absence)			(0.40-2.49)		(0.62-1.84)		(0.54-1.72)
Social position of origin (Reference: I&II)							
III non-manual	Ref	--	0.68 (0.41-1.13)	--	1.14 (0.84-1.54)	--	0.95 (0.68-1.33)
III manual	Ref	--	0.81 (0.56-1.18)	--	1.27 (1.00-1.62)	--	1.36 (1.05-1.76)
IV&V + no male head	Ref	--	0.94 (0.61-1.42)	--	1.37 (1.05-1.79)	--	1.46 (1.10-1.93)

For peer review only

Table 3: Direct effects of childhood exposures on adult respiratory health independent of adult smoking, indicated by Wald test results

	Phlegm only χ^2(DF)	Cough only χ^2(DF)	Phlegm + Cough χ^2(DF)
Smoking status at age 10	2.89(2)	0.99(1)	7.22(1)**
Numbers of adult smokers in the household at age 10	4.37(1)*	6.12(2)*	6.09(2)*
Household dampness at age 10	5.87(2)	2.83(2)	28.79(2)***

Note: * p<0.05, ** p<0.01, ***p <0.001

For peer review only

References

1. Department of Health. Healthy lives, healthy people: Our strategy for public health in England. London: Department of Health, 2010:1-95.

2. Gray LA, Leyland AH, Benzeval M, Watt GC. Explaining the social patterning of lung function in adulthood at different ages: the roles of childhood precursors, health behaviours and environmental factors. *J Epidemiol Community Health* 2013.

3. Guerra S, Stern DA, Zhou M, Sherrill DL, Wright AL, Morgan WJ, et al. Combined effects of parental and active smoking on early lung function deficits: a prospective study from birth to age 26 years. *Thorax* 2013.

4. Svanes C, Omenaas E, Jarvis D, Chinn S, Gulsvik A, Burney P. Parental smoking in childhood and adult obstructive lung disease: results from the European Community Respiratory Health Survey. *Thorax* 2004;59(4):295-302.

5. Beyer D, Mitfessel H, Gillissen A. Maternal smoking promotes chronic obstructive lung disease in the offspring as adults. *Eur. J. Med. Res.* 2009;14 Suppl 4:27-31.

6. Bartley M, Kelly Y, Sacker A. Early life financial adversity and respiratory function in midlife: a prospective birth cohort study. *Am J Epidemiol* 2012;175(1):33-42.

7. Mann SL, Wadsworth ME, Colley JR. Accumulation of factors influencing respiratory illness in members of a national birth cohort and their offspring. *J Epidemiol Community Health* 1992;46(3):286-92.

8. Jackson B, Kubzansky LD, Cohen S, Weiss S, Wright RJ. A matter of life and breath: childhood socioeconomic status is related to young adult pulmonary function in the CARDIA study. *Int J Epidemiol* 2004;33(2):271-8.

9. Kilpelainen M, Terho EO, Helenius H, Koskenvuo M. Home dampness, current allergic diseases, and respiratory infections among young adults. *Thorax* 2001;56(6):462-7.

For peer review only

10. Norback D, Zock JP, Plana E, Heinrich J, Svanes C, Sunyer J, et al. Mould and dampness in dwelling places, and onset of asthma: the population-based cohort ECRHS. *Occup Environ Med* 2013;70(5):325-31.

11. Pirastu R, Bellu C, Greco P, Pelosi U, Pistelli R, Accetta G, et al. Indoor exposure to environmental tobacco smoke and dampness: respiratory symptoms in Sardinian children--DRIAS study. *Environ. Res.* 2009;109(1):59-65.

12. Simoni M, Lombardi E, Berti G, Rusconi F, La Grutta S, Piffer S, et al. Mould/dampness exposure at home is associated with respiratory disorders in Italian children and adolescents: the SIDRIA-2 Study. *Occup Environ Med* 2005;62(9):616-22.

13. Zock JP, Jarvis D, Luczynska C, Sunyer J, Burney P. Housing characteristics, reported mold exposure, and asthma in the European Community Respiratory Health Survey. *J. Allergy Clin. Immunol.* 2002;110(2):285-92.

14. Weinmayr G, Gehring U, Genuneit J, Buchele G, Kleiner A, Siebers R, et al. Dampness and moulds in relation to respiratory and allergic symptoms in children: results from Phase Two of the International Study of Asthma and Allergies in Childhood (ISAAC Phase Two). *Clin. Exp. Allergy* 2013;43(7):762-74.

15. Tennant PW, Gibson GJ, Parker L, Pearce MS. Childhood respiratory illness and lung function at ages 14 and 50 years: childhood respiratory illness and lung function. *Chest* 2010;137(1):146-55.

16. Elliott J, Shepherd P. Cohort Profile: 1970 British Birth Cohort (BCS70). *International Journal of Epidemiology* 2006;35 836-43.

17. Chamberlain R, St Mary's Hospital, Chamberlain G, Queen Charotte's Hospital, University of London Institute of Education Centre for Longitudinal Studies. 1970 Birth Cohort Study: Birth and 22-month Subsample, 1970-1972. 3rd edition ed: UK Data Archive University of Essex Colchester, 2013.

For peer review only

18. Butler N, Bynner JM, University of London Institute of Education Centre for Longitudinal Studies. 1970 British Birth Cohort Study: Ten-Year Follow-up, 1980. 4th edition ed. Colchester: UK data Archive University of Essex Colchester, 2013.

19. University of London Institute of Education Centre for Longitudinal Studies. 1970 British Birth Cohort Study: Twenty-Nine-Year Follow-up, 1999-2000 [computer file]. 3rd Edition. Colchester, Essex: UK Data Archive University of Essex [distributor].

20. Shepherd P. 1979 British Cohort Study Ethical Review and consent. London: Centre for Longitudinal Studies, 2012:1-14.

21. Power C, Elliott J. Cohort profile: 1958 British birth cohort (National Child Development Study). *International Journal of Epidemiology* 2006;35(1):34-41.

22. Strachan DP, Anderson HR, Bland JM, Peckham C. Asthma as a link between chest illness in childhood and chronic cough and phlegm in young adults. *Br. Med. J. (Clin. Res. Ed).* 1988;296(6626):890-3.

23. Power C, Hypponen E, Smith GD. Socioeconomic position in childhood and early adult life and risk of mortality: a prospective study of the mothers of the 1958 British birth cohort. *Am J Public Health* 2005;95(8):1396-402.

24. Stata Statistical Software: Release 12 [program]. V12.0 version. College Station, TX: StataCorp LP, 2011.

25. Sahlberg B, Norback D, Wieslander G, Gislason T, Janson C. Onset of mucosal, dermal, and general symptoms in relation to biomarkers and exposures in the dwelling: a cohort study from 1992 to 2002. *Indoor Air* 2012;22(4):331-8.

For peer review only

STROBE Statement—checklist of items that should be included in reports of observational studies

	Item No	Recommendation
**Title and abstract**	1	(*a*) Indicate the study's design with a commonly used term in the title or the abstract
		(*b*) Provide in the abstract an informative and balanced summary of what was done and what was found
**Introduction**		
Background/rationale	2	Explain the scientific background and rationale for the investigation being reported
Objectives	3	State specific objectives, including any prespecified hypotheses
**Methods**		
Study design	4	Present key elements of study design early in the paper
Setting	5	Describe the setting, locations, and relevant dates, including periods of recruitment, exposure, follow-up, and data collection
Participants	6	(*a*) *Cohort study*—Give the eligibility criteria, and the sources and methods of selection of participants. Describe methods of follow-up<br>*Case-control study*—Give the eligibility criteria, and the sources and methods of case ascertainment and control selection. Give the rationale for the choice of cases and controls<br>*Cross-sectional study*—Give the eligibility criteria, and the sources and methods of selection of participants
		(*b*) *Cohort study*—For matched studies, give matching criteria and number of exposed and unexposed<br>*Case-control study*—For matched studies, give matching criteria and the number of controls per case
Variables	7	Clearly define all outcomes, exposures, predictors, potential confounders, and effect modifiers. Give diagnostic criteria, if applicable
Data sources/ measurement	8*	For each variable of interest, give sources of data and details of methods of assessment (measurement). Describe comparability of assessment methods if there is more than one group
Bias	9	Describe any efforts to address potential sources of bias
Study size	10	Explain how the study size was arrived at
Quantitative variables	11	Explain how quantitative variables were handled in the analyses. If applicable, describe which groupings were chosen and why
Statistical methods	12	(*a*) Describe all statistical methods, including those used to control for confounding
		(*b*) Describe any methods used to examine subgroups and interactions
		(*c*) Explain how missing data were addressed
		(*d*) *Cohort study*—If applicable, explain how loss to follow-up was addressed<br>*Case-control study*—If applicable, explain how matching of cases and controls was addressed<br>*Cross-sectional study*—If applicable, describe analytical methods taking account of sampling strategy
		(*e*) Describe any sensitivity analyses

Continued on next page

For peer review only

**Results**		
Participants	13*	(a) Report numbers of individuals at each stage of study—eg numbers potentially eligible, examined for eligibility, confirmed eligible, included in the study, completing follow-up, and analysed
		(b) Give reasons for non-participation at each stage
		(c) Consider use of a flow diagram
Descriptive data	14*	(a) Give characteristics of study participants (eg demographic, clinical, social) and information on exposures and potential confounders
		(b) Indicate number of participants with missing data for each variable of interest
		(c) *Cohort study*—Summarise follow-up time (eg, average and total amount)
Outcome data	15*	*Cohort study*—Report numbers of outcome events or summary measures over time
		Case-control study—Report numbers in each exposure category, or summary measures of exposure
		Cross-sectional study—Report numbers of outcome events or summary measures
Main results	16	(*a*) Give unadjusted estimates and, if applicable, confounder-adjusted estimates and their precision (eg, 95% confidence interval). Make clear which confounders were adjusted for and why they were included
		(*b*) Report category boundaries when continuous variables were categorized
		(*c*) If relevant, consider translating estimates of relative risk into absolute risk for a meaningful time period
Other analyses	17	Report other analyses done—eg analyses of subgroups and interactions, and sensitivity analyses
**Discussion**		
Key results	18	Summarise key results with reference to study objectives
Limitations	19	Discuss limitations of the study, taking into account sources of potential bias or imprecision. Discuss both direction and magnitude of any potential bias
Interpretation	20	Give a cautious overall interpretation of results considering objectives, limitations, multiplicity of analyses, results from similar studies, and other relevant evidence
Generalisability	21	Discuss the generalisability (external validity) of the study results
**Other information**		
Funding	22	Give the source of funding and the role of the funders for the present study and, if applicable, for the original study on which the present article is based

*Give information separately for cases and controls in case-control studies and, if applicable, for exposed and unexposed groups in cohort and cross-sectional studies.

Note: An Explanation and Elaboration article discusses each checklist item and gives methodological background and published examples of transparent reporting. The STROBE checklist is best used in conjunction with this article (freely available on the Web sites of PLoS Medicine at http://www.plosmedicine.org/, Annals of Internal Medicine at http://www.annals.org/, and Epidemiology at http://www.epidem.com/). Information on the STROBE Initiative is available at www.strobe-statement.org.

For peer review only

Critical role of smoking and household dampness during childhood for adult phlegm and cough: A research example from a prospective cohort study in Great Britain

Journal:	*BMJ Open*
Manuscript ID:	bmjopen-2014-004807.R1
Article Type:	Research
Date Submitted by the Author:	12-Mar-2014

Complete List of Authors:	Cable, Noriko; UCL, Epidemiology and Public Health<br>Bartley, Mel; UCL, Epidemiology and Public Health<br>Kelly, Yvonne; UCL, Epidemiology and Public Health<br>Sato, Yuki; National Institute for Environmental Studies, Centre for Environmental Health Sciences<br>Sacker, Amanda; UCL, Epidemiology and Public Health

**Primary Subject Heading**:	Epidemiology
Secondary Subject Heading:	Respiratory medicine, Public health
Keywords:	BCS70, household dampness, adult respiratory health, Life course study

SCHOLARONE™ Manuscripts

For peer review only

Critical role of smoking and household dampness during childhood **for** adult **phlegm and cough**: A research example from **a prospective cohort study in Great Britain**

Authors: Noriko Cable[1], Yvonne Kelly[1], Mel Bartley[1], Yuki Sato[2], Amanda Sacker[1]

[1]Research Department of Epidemiology and Public Health, University College London

1-19 Torrington Place, London WC1E 6BT United Kingdom: Noriko Cable PhD, Senior Research Fellow, Yvonne Kelly PhD, Professor of Life course Epidemiology, Mel Bartley PhD, Professor of Medical Sociology, Amanda Sacker PhD, Professor of Lifecourse Studies

[2]Centre for Environmental Health Sciences, National Institute for Environmental Studies, 16-2 Onogawa, Tsukuba-city, Ibaragi 305-8506, Japan: Yuki Sato PhD, Researcher

Correspondence to: n.cable@ucl.ac.uk

For peer review only

Strength and limitations of the study

- This study uses a large sample with the prospective cohort design, allowing to examined the longitudinal associations between childhood exposures (parental and childhood smoking, household dampness) and adult phlegm and cough without introducing recall bias
- Confounders (sex, childhood SES, respiratory difficulties at birth, childhood phlegm and cough) and adult smoking were accounted to identify significant associations between exposures at age 10 (childhood smoking, parental smoking, and household dampness) and adult phlegm and cough at age 29.
- **The outcome variable that is patterns of two respiratory symptoms (phlegm and cough) was derived extracted from a well-established questionnaire and not indicative of a particular respiratory disease or lung function**.

For peer review only

Abstract

Objective: To examine independent associations between childhood exposures to smoking and housing dampness and **phlegm and cough in adulthood**.

Design: A prospective cohort study.

Participants: 7,320 of the British cohort who were born during one week in 1970 and had complete data for childhood and adult information.

Main outcome measures: Experiences of phlegm and coughing over the previous three months were assessed using questions from the MRC **Questionnaire on respiratory symptoms** when the cohort participants were 29 years of age. Four response patterns (no symptoms, phlegm only, cough only, both symptoms present) were created based on the responses to these questions.

Results: Childhood smoking and exposure to **marked** household dampness at age 10 were associated with **phlegm (childhood smoking: RRR= 1.45, 95%CI=1.02-2.05; dampness: RRR=2.05, 95%CI=1.07-3.91) and co-occurring cough and phlegm (childhood smoking: RRR=1.35. 95%CI=1.08-1.67; dampness: RRR= 2.73, 95%CI=1.88-3.99),** while exposure to **two or more** adult smokers in the household was associated with cough-related symptoms **(cough only: RRR=1.28, 95%CI=1.04-1.58; phlegm and cough: RRR=1.32, 95%CI=1.06-1.64).** These associations were independent from adult smoking, childhood **phlegm and cough**, early social background, and sex. Current smoking at age 29 **contributed to all symptom patters; however, a substantial association between household dampness and co-occurring phlegm and cough suggest long term detrimental effects of childhood environmental exposures.**

Conclusions: Our findings give support to current public health interventions for adult smoking and raise concerns about the long-term effects of a damp home environment on the respiratory health of children.

For peer review only

Introduction

Smoking cessation has been supported as the main preventive measure to reduce smoking-related illnesses and mortality[1]. Previous findings have suggested additional risk factors for adult respiratory health. There are longitudinal links between exposure to parental smoking[2-5] or social disadvantage[6-8] during childhood and adult respiratory health, while research evidence to support the association between indoor dampness and respiratory health is mainly cross-sectional[9-14].

Examining the life course respiratory health of the birth cohort of Britons born in 1946, Mann, Wadsworth and Colley[7] reported that the greatest contribution to adult respiratory health was from proximal risk factors such as adult smoking rather than from distant risk factors such as child respiratory health or social disadvantage. Since adult respiratory health is **likely to be** determined by child respiratory health[15], additional longitudinal effects of childhood exposures, such as smoking and exposure to household damp on adult respiratory health may be small.

However, not many studies have used repeated measures **of** respiratory health **in both childhood and adulthood**. The contribution of childhood exposures to adult respiratory health can be better estimated if a similar respiratory measure is used to assess both child and adult respiratory health. Here we use respiratory symptoms of phlegm and cough occurring in childhood and in adulthood to examine the degree of contribution to **these** adult respiratory **symptoms** from exposure to smoking and household damp during childhood. We used a British cohort who were born in 1970 and examined **this** hypothesis.

1. Household dampness, exposure to adult cigarette smoke during childhood, and childhood smoking will have long-term associations with adult respiratory **symptoms** over and above childhood respiratory **symptoms** and smoking in adulthood.

For peer review only

Method:

Study design, setting and participants

We used information obtained from a prospective cohort study, the 1970 British Cohort Study (BCS70). Detailed information about research design and collected data is given by Elliot and Shepherd[16]. BCS70 targets British residents who were born during one week in 1970; data have been collected regularly across their life course. We used datasets collected at birth[17], at age 10[18] and at age 29[19] to extract study variables. In this study, 7,320 available cases which contain all of the study information were used for the analyses.

Ethical approvals of the study

Data collection at birth and at age 10 was reviewed internally, while ethical approval from the London Multi-centre Research Ethics Committee was obtained for the age 29 survey[20].

Explanatory factors

We used three childhood exposures (child's own smoking, number of adult smokers in the household, household dampness) as explanatory factors. Information about these factors was collected when the cohort children were 10 years of age. A cohort child's own smoking status was indicated by the response to the question: 'Have you tried a cigarette?' Children who had tried a cigarette were coded as 1 as opposed to those who had not yet experimented with smoking (=0). The number of adult smokers in the household was derived through the response about the smoking status of the parents and other adults in the household obtained through a parental interview. This response was tabulated into three categories: 0 for no adult smokers, 1 for one adult, and 2 for two or more adults. **In our study a smoking child could have been exposed to adult smokers at home; however our interest is to assess the role of each factor separately, thus we treated these factors independently.**

In addition, the degree of household dampness was addressed via this parental interview, asking them to rate **the present state of** household dampness as: none, slight, moderate, or

For peer review only - http://bmjopen.bmj.com/site/about/guidelines.xhtml

marked. Only a few people responded that their house was ‘moderately’ damp; therefore, this response was included in the category of ‘slight’ dampness.

Adulthood factor

Information about adult smoking was collected when the cohort participants were 29 years of age. Their smoking status was indicated as 0 for current non-smokers and 1 for current smokers.

Outcome variable

A pattern of **co-occurring** phlegm and cough **(no symptoms, phlegm only, cough only, phlegm and cough) in adulthood** was the outcome of this study. Cohort participants were asked to respond whether they **usually** brought up phlegm or experienced **coughing** in the morning, during the day or at night in the winter when they were 29 years old. These questions were from the Medical Research Council Questionnaire **on respiratory symptoms** and have been widely used, including in the previous cohort study (i.e. National Child Developmental Study). [21] We adapted the method used by Strachan et al.[22] and created a variable to indicate presence of each respiratory symptom (=phlegm or cough), which cohort participants might have experienced at any time of day. After that we derived a variable with four response patterns of adult respiratory symptoms by combining the response to each variable **obtained at age 29**: 0 no respiratory symptoms, 1 phlegm only, 2 cough only, and 3 both phlegm and cough.

Confounders

We controlled for childhood **phlegm and cough**, sex, paternal social position. **Presence of** phlegm (0=no, 1=yes) or cough (0=no, 1=yes) when the cohort children were 10 years old **was used**. These variables were created through the responses (no, yes but for less than 3 months a year, yes for 3 months or more a year, not known) to the questions: ‘Does the study child usually cough/bring up phlegm first thing in the morning/during the day or at night?’

For peer review only

These questions are similar to ones from the MRC Questionnaire, with the omission of the phrase ‘in the winter’. A positive response to a particular respiratory question was treated as presence of the particular respiratory symptom (phlegm or cough), while the response ‘no’ was treated as absence of that respiratory symptom. The response ‘not known’ was regarded as missing and excluded from the analyses. We also included the presence of respiratory difficulties, including respiratory distress syndrome, at the birth of the cohort children (0=absent, 1=present) in the model.

Parental social position was indicated by paternal occupational position at the birth of the cohort child. We adapted the approach by Power et al.[23], classifying the Registrar General’s Social Classes of the cohort children’s father as: 1 – professional or managerial (I& II); 2 – skilled non-manual (III non-manual); 3 – skilled manual (III manual); 4 – partly skilled or unskilled (IV&V) and children who were born in a mother-only household. Midwives provided the information relevant to the birth of the cohort children, while the presence of phlegm or cough was assessed via the parental interview.

Statistical analysis

Multinomial logistic regression was used to identify associations between explanatory factors and patterns of adult respiratory symptoms (phlegm, cough, or both). We took those who did not have any respiratory symptoms as a reference category. **First we entered each explanatory variable in separate models, adjusting for sex. In a final model estimates for all exposures are estimated after adjustment for each other and the confounders.** In our study men and women were analysed together, controlling for the effect of sex. All statistical analyses were performed using Stata SE v12[24].

Results

Study characteristics

Approximately 2.5% of mothers of the cohort children reported that their children brought up phlegm at age 10, while 11% **reported** cough (N=7320). **C**o-occurring symptoms of phlegm and cough **was reported for 8.7% of participants** at age 29, while 2.9% **reported** phlegm only and 9.2% cough **only**. Assessing respiratory symptoms across the life course, **only a** small proportion of participants continuously experienced respiratory symptoms at both times (Table 1).

According to maternal report**s**, less than half of the cohort children grew up in a smoke-free domestic environment, while household dampness of any degree was observed in around 17% of the **homes** of the cohort children. Many of the cohort participants did not smoke at 10 years of age, but around half of the cohort participants were current smokers at age 29. Demographically 48% of the participants were men and 30% of the participants had fathers with non-manual occupations. **Most** of the cohort participants did not experience respiratory difficulties when they were born.

Long-term associations between childhood factors and adult ***phlegm or cough***

The results of multinomial logistic regression showed childhood factors were independently and specifically associated with **patterns of** adult respiratory **symptoms (phlegm or cough)** (Table 2). The relative risk ratio (RRR) for childhood smoking was 1.45 (95%CI=1.02-2.05) for phlegm only and was 1.35 (95%CI=1.08-1.67) for co-occurring phlegm and cough. Growing up in a markedly damp house showed a similar, but stronger association with phlegm only (RRR=2.05, 95%CI=1.07-3.91) and co-occurring phlegm and cough (RRR=2.73, 95%CI=1.88-3.99).

Having adult smokers in the house when the cohort child was 10 years old was positively associated- with all patterns of respiratory symptoms; however, having two or more adult

smokers were significantly associated with adult cohort participants' respiratory symptoms that were cough only (RRR=1.28, 95%CI=1.04-1.58) or co-occurrence of phlegm and cough (RRR=1.32, 95%CI=1.06-1.64). These associations **were** independent of adult smoking and confounders. Adult smoking at age 29 was significantly associated with all of the respiratory response patterns.

Discussion

Key results

Using a British cohort born in 1970, we showed significant longitudinal associations between childhood exposures (child's own smoking, two or more adult smokers in the house, and marked household dampness) and patterns of adult **phlegm or cough**. These were independent of **presence of phlegm or cough at age 10**, adult smoking status, sex and social position of origin. Although the contribution from childhood exposures to adult respiratory **symptom patterns (phlegm, cough or both**) was relatively small compared to adult smoking, household dampness at age 10 showed a substantial contribution to co-occurring phlegm and cough at age 29.

Strengths and limitations

This study benefits from a substantially large sample size with a prospective design which limits recall bias. Questions from the MRC Questionnaire were used to identify the patterns of respiratory symptoms (either phlegm or cough, or both symptoms present) at age 29, rather than the presence of respiratory diseases. We consider that identifying respiratory symptoms of phlegm or cough at age 10, using similar questions to the adult questionnaire, enabled us to estimate associations between childhood exposures and adult respiratory symptom **patterns (phlegm, cough or both)** with considerable accuracy. The omission of the words 'in the

For peer review only

winter' in the child version of the respiratory questions, however, may have over-identified childhood cases of phlegm or cough. In addition, our findings relating to childhood smoking status should be interpreted carefully as they were obtained via self-report. An objective measure of lung function is not available in any sweep of the BCS70 data which is also a limitation to our study.

Information about the duration of exposure to indoor dampness is not available**. In their reports published in 2005, the World Health Organisation[25] recommends detailed and objective assessment of indoor dampness, such as using a trained assessor or measurements of levels of humidity**. In our study we used parental report on household dampness when the cohort children were 10 year old of age (i.e. 1980). This was the only available measure of household dampness. **However, finding a longitudinal association between marked dampness and phlegm related adult respiratory symptoms suggests** that exposure to severe indoor dampness may have irritated the respiratory system of the cohort children, even though they were asymptomatic at that time. **We are unable to account for air pollution, which could affect indoor air quality via ventilation[25] because the information is not available. Further studies with detailed assessments of household dampness and air quality are needed.**

Possible explanations and comparison with previous studies

We found that experimenting with smoking and exposures to marked household dampness at 10 years of age were longitudinally associated with phlegm-related symptoms at age 29, while having two or more adult smokers in the household showed detrimental associations with cough-related symptoms at the same age. These longitudinal associations are independent of previous **phlegm or cough**, adult smoking, and other confounders. It is worth noting that nearly 80% of the cohort participants (n=11,648) did not have any respiratory

For peer review only

symptoms at age 10 to start with, yet 20% of these participants had developed respiratory symptoms, phlegm or cough by age 29. This is somewhat similar to the findings by Guerra et al.[3] that lung function became markedly poorer at age 26 only when participants were exposed to both parental smoking at their birth and were **smokers themselves**. It is possible that **development of phlegm or cough** may not coincide with earlier exposures to smoking or household dampness. Our findings also add to the existing findings **that** link parental smoking and respiratory symptoms in adulthood [2 4] and indicate that the mode (active vs. second-hand) of childhood exposure to smoking may affect respiratory systems differently. Our finding linking household dampness with presence of phlegm in adulthood is similar to the findings by Sahlberg et al.[26] that adults exposed to household dampness showed increased occurrence of mucosal symptoms 10 years **later**. **Our finding is also similar to the meta analyses results by Mendell et al.[27], showing a substantial association between indoor dampness and various respiratory symptoms, including cough.**

Our finding supports longitudinal associations between childhood exposures and adult respiratory **symptoms** similarly to the study by Mann et al.[7] **We are** the first to show a substantial contribution of childhood exposure to household dampness to co-occurring phlegm and cough at age 29.

Implications and conclusions

In summary, we **found** support **for** longitudinal associations between childhood exposures to smoking and household dampness, and **patterns of** adult respiratory **symptoms**. Although **adult smoking contributed to all of** adult respiratory **symptom patterns,** exposure to **marked** household dampness during childhood showed a substantial contribution to co-occurring phlegm and cough. Our findings support current public health interventions to reduce adult smoking, but also indicate that the management of childhood risk factors such as

For peer review only

exposure to smoke (active or second-hand) and household dampness can be a way to prevent adults experiencing poor respiratory health.

For peer review only

Footnotes:

Contributors: NC planned the study, analysed the data, and participated in writing and discussion. AS advised statistical analyses and participated in writing and discussion. MB, YK and YS participated in writing and discussion.

Funding information: This work has funded through the Economic and Social Research Council's International Centre for Life Course Studies in Society and Health (ES/J019119/1).

Conflicts of Interests: None declared

Ethical approval: BCS70 data used in this study were managed by the Centre for Longitudinal Study and accessible via the UK data archive. The data do not contain identifiable information and academic use of the BCS70 data does not require ethical approvals.

Data sharing statement: BCS70 data used in this study have been managed by the Centre for Longitudinal Study and are accessible via the UK data archive. The statistical code for derived variables is available from the correspondence author.

For peer review only

Table 1. Description (%) of explanatory and confounding variables by respiratory conditions (no symptoms, phlegm only, cough only, phlegm and cough) at age 29 (N=7,320)

	No symptoms % (n=5792)	Phlegm % (n=214)	Cough % (n=675)	Phlegm + Cough % (n=639)
**Explanatory variables**				
Smoking status at age 10				
Never	88.09	78.97	84.74	79.50
Smoked	11.92	21.12	15.26	20.50
Household dampness at age 10				
No dampness	84.34	83.18	84.15	76.37
Slight to moderate	13.33	11.68	12.40	16.91
Marked	2.33	5.14	3.41	6.73
Numbers of adult smokers in the household at age 10				
Zero	36.08	28.50	29.93	26.60
One	36.31	38.32	35.83	38.50
Two or more	27.61	33.18	34.22	34.90
Smoking status at age 29				
Current smoker	49.64	70.56	70.81	80.44
**Confounders**				
Phlegm at age 10				
present	2.05	5.61	4.00	4.38
Cough at age 10				
Present	9.94	14.49	14.37	16.28
Respiratory difficulties at birth				
Present	2.14	2.34	2.22	2.19
Social position of origin				
**Professional & managerial**	18.42	20.56	14.67	13.77
**Skilled non-manual**	14.50	11.21	13.04	10.33
**Skilled manual**	45.51	42.99	46.96	48.36
**Non-skilled manual + no male head**	21.56	25.23	25.33	27.54
Sex				
Male	45.98	67.76	45.63	63.85
Female	54.02	32.24	54.37	36.15

For peer review only

Table 2. Associations between child and adult risk factors and adult respiratory conditions expressed **as** relative risk ratios (RRR) and 95% **confidence intervals in parentheses** obtained by multinomial logistic regression (N=7320)

	No symptoms (n=5792)	Phlegm only (n=214)		Cough only (n=675)		Phlegm + Cough (n=639)	
		Bivariate[a]	Fully adjusted[b]	Bivariate[a]	Fully adjusted[b]	Bivariate[a]	Fully adjusted[b]
**Explanatory factors**							
Smoking status at age 10 (Reference: Not smoked)	Ref	**1.74 (1.24-2.45)**	1.45 (1.02-2.05)	**1.34 (1.07-1.68)**	1.12 (0.89-1.41)	**1.72 (1.40-2.13)**	1.35 (1.08-1.67)
Household dampness at age 10 (Reference: no dampness)							
Slight to moderate	Ref	**0.89 (0.58-1.37)**	0.82 (0.54-1.27)	**0.94 (0.74-1.19)**	0.85 (0.67-1.09)	**1.40 (1.12-1.75)**	1.24 (0.99-1.56)
Marked	Ref	**2.33 (1.23-4.41)**	2.05 (1.07-3.91)	**1.46 (0.93-2.30)**	1.26 (0.80-1.99)	**3.30 (2.31-4.73)**	2.73 (1.88-3.99)
Numbers of adult smokers in the household at age 10 (reference: Zero)							
One	Ref	**1.35 (0.96-1.90)**	1.23 (0.88-1.74)	**1.19 (0.98-1.45)**	1.07 (0.87-1.30)	**1.45 (1.18-1.78)**	1.20 (0.97-1.49)
Two or more	Ref	**1.54 (1.09-2.19)**	1.36 (0.95-1.95)	**1.49 (1.22-1.82)**	1.28 (1.04-1.58)	**1.74 (1.41-2.15)**	1.32 (1.06-1.64)
Current smoker at age 29 (Reference: Non-smoking)	Ref	**2.40 (1.78-3.24)**	2.29 (1.69-3.09)	**2.46 (2.07-2.93)**	2.41 (2.02-2.88)	**4.13 (3.37-5.06)**	3.95 (3.22-4.85)
**Confounders**							
Phlegm present at age 10 (Reference: Absent)	Ref	--	2.62 (1.28-5.39)	--	1.60 (0.99-2.58)	--	1.63 (1.01-2.64)
Cough present at age 10 (Reference: Absent)	Ref	--	1.14 (0.72-1.81)	--	1.34 (1.03-1.74)	--	1.45 (1.12-1.89)
Sex (Reference: Men)	Ref	--	0.42 (0.31-0.56)	--	1.03 (0.87-1.21)	--	0.49 (0.41-0.59)
Respiratory difficulties at birth	Ref	--	1.00	--	1.06	--	0.97

For peer review only

(Reference: Absence)			(0.40-2.49)		(0.62-1.84)		(0.54-1.72)
Social position of origin (Reference: **Professional and managerial**)							
**Skilled non-manual**	Ref	--	0.68 (0.41-1.13)	--	1.14 (0.84-1.54)	--	0.95 (0.68-1.33)
**Skilled manual**	Ref	--	0.81 (0.56-1.18)	--	1.27 (1.00-1.62)	--	1.36 (1.05-1.76)
**Non-skilled manual** + no male head	Ref	--	0.94 (0.61-1.42)	--	1.37 (1.05-1.79)	--	1.46 (1.10-1.93)

[a] **Estimates are adjusted for sex**

[b] **Estimates for explanatory factors are adjusted for sex, confounders and other exposures**

For peer review only

References

1. Department of Health. Healthy lives, healthy people: Our strategy for public health in England. London: Department of Health, 2010:1-95.

2. Gray LA, Leyland AH, Benzeval M, Watt GC. Explaining the social patterning of lung function in adulthood at different ages: the roles of childhood precursors, health behaviours and environmental factors. *J Epidemiol Community Health* 2013;67(11):905-11.

3. Guerra S, Stern DA, Zhou M, Sherrill DL, Wright AL, Morgan WJ, et al. Combined effects of parental and active smoking on early lung function deficits: a prospective study from birth to age 26 years. *Thorax* 2013.

4. Svanes C, Omenaas E, Jarvis D, Chinn S, Gulsvik A, Burney P. Parental smoking in childhood and adult obstructive lung disease: results from the European Community Respiratory Health Survey. *Thorax* 2004;59(4):295-302.

5. Beyer D, Mitfessel H, Gillissen A. Maternal smoking promotes chronic obstructive lung disease in the offspring as adults. *Eur. J. Med. Res.* 2009;14 Suppl 4:27-31.

6. Bartley M, Kelly Y, Sacker A. Early life financial adversity and respiratory function in midlife: a prospective birth cohort study. *Am J Epidemiol* 2012;175(1):33-42.

7. Mann SL, Wadsworth ME, Colley JR. Accumulation of factors influencing respiratory illness in members of a national birth cohort and their offspring. *J Epidemiol Community Health* 1992;46(3):286-92.

8. Jackson B, Kubzansky LD, Cohen S, Weiss S, Wright RJ. A matter of life and breath: childhood socioeconomic status is related to young adult pulmonary function in the CARDIA study. *Int J Epidemiol* 2004;33(2):271-8.

9. Kilpelainen M, Terho EO, Helenius H, Koskenvuo M. Home dampness, current allergic diseases, and respiratory infections among young adults. *Thorax* 2001;56(6):462-7.

For peer review only

10. Norback D, Zock JP, Plana E, Heinrich J, Svanes C, Sunyer J, et al. Mould and dampness in dwelling places, and onset of asthma: the population-based cohort ECRHS. *Occup Environ Med* 2013;70(5):325-31.

11. Pirastu R, Bellu C, Greco P, Pelosi U, Pistelli R, Accetta G, et al. Indoor exposure to environmental tobacco smoke and dampness: respiratory symptoms in Sardinian children--DRIAS study. *Environ. Res.* 2009;109(1):59-65.

12. Simoni M, Lombardi E, Berti G, Rusconi F, La Grutta S, Piffer S, et al. Mould/dampness exposure at home is associated with respiratory disorders in Italian children and adolescents: the SIDRIA-2 Study. *Occup Environ Med* 2005;62(9):616-22.

13. Zock JP, Jarvis D, Luczynska C, Sunyer J, Burney P. Housing characteristics, reported mold exposure, and asthma in the European Community Respiratory Health Survey. *J. Allergy Clin. Immunol.* 2002;110(2):285-92.

14. Weinmayr G, Gehring U, Genuneit J, Buchele G, Kleiner A, Siebers R, et al. Dampness and moulds in relation to respiratory and allergic symptoms in children: results from Phase Two of the International Study of Asthma and Allergies in Childhood (ISAAC Phase Two). *Clin. Exp. Allergy* 2013;43(7):762-74.

15. Tennant PW, Gibson GJ, Parker L, Pearce MS. Childhood respiratory illness and lung function at ages 14 and 50 years: childhood respiratory illness and lung function. *Chest* 2010;137(1):146-55.

16. Elliott J, Shepherd P. Cohort Profile: 1970 British Birth Cohort (BCS70). *International Journal of Epidemiology* 2006;35 836-43.

17. Chamberlain R, Chamberlain G, University of London Institute of Education Centre for Longitudinal Studies. 1970 Birth Cohort Study: Birth and 22-month Subsample, 1970-1972. 3rd edition ed: UK Data Archive University of Essex Colchester, 2013.

For peer review only

18. Butler N, Bynner JM, University of London Institute of Education Centre for Longitudinal Studies. 1970 British Birth Cohort Study: Ten-Year Follow-up, 1980. 4th edition ed. Colchester: UK data Archive University of Essex Colchester, 2013.

19. University of London Institute of Education Centre for Longitudinal Studies. 1970 British Birth Cohort Study: Twenty-Nine-Year Follow-up, 1999-2000. 3rd edition ed. Colchester: UK Data Archive University of Essex Colchester, 2013.

20. Shepherd P. 1979 British Cohort Study Ethical Review and consent. London: Centre for Longitudinal Studies, 2012:1-14.

21. Power C, Elliott J. Cohort profile: 1958 British birth cohort (National Child Development Study). *International Journal of Epidemiology* 2006;35(1):34-41.

22. Strachan DP, Anderson HR, Bland JM, Peckham C. Asthma as a link between chest illness in childhood and chronic cough and phlegm in young adults. *Br. Med. J. (Clin. Res. Ed).* 1988;296(6626):890-3.

23. Howe LD, Hargreaves JR, Ploubidis GB, De Stavola BL, Huttly SR. Subjective measures of socio-economic position and the wealth index: a comparative analysis. *Health Policy Plan.* 2011;26(3):223-32.

24. Stata Statistical Software: Release 12 [program]. V12.0 version. College Station, TX: StataCorp LP, 2011.

25. World Health Organization. WHO guidelines for indoodr air quality: dampness and mould. In: Heseltine E, Rosen J, editors. Copenhagen, 2009:1-248.

26. Sahlberg B, Norback D, Wieslander G, Gislason T, Janson C. Onset of mucosal, dermal, and general symptoms in relation to biomarkers and exposures in the dwelling: a cohort study from 1992 to 2002. *Indoor Air* 2012;22(4):331-8.

For peer review only

27. Mendell MJ, Mirer AG, Cheung K, Tong M, Douwes J. Respiratory and Allergic Health Effects of Dampness, Mold, and Dampness-Related Agents: A Review of the Epidemiologic Evidence. *Environ. Health Perspect.* 2010;119:748-56.

For peer review only

Critical role of smoking and household dampness during childhood **for** adult **phlegm and cough**: A research example from **a prospective cohort study in Great Britain**

Authors: Noriko Cable[1], Yvonne Kelly[1], Mel Bartley[1], Yuki Sato[2], Amanda Sacker[1]

[1]Research Department of Epidemiology and Public Health, University College London

1-19 Torrington Place, London WC1E 6BT United Kingdom: Noriko Cable PhD, Senior Research Fellow, Yvonne Kelly PhD, Professor of Life course Epidemiology, Mel Bartley PhD, Professor of Medical Sociology, Amanda Sacker PhD, Professor of Lifecourse Studies

[2]Centre for Environmental Health Sciences, National Institute for Environmental Studies, 16-2 Onogawa, Tsukuba-city, Ibaragi 305-8506, Japan: Yuki Sato PhD, Researcher

Correspondence to: n.cable@ucl.ac.uk

For peer review only

Strength and limitations of the study

- This study uses a large sample with the prospective cohort design, allowing to examined the longitudinal associations between childhood exposures (parental and childhood smoking, household dampness) and adult phlegm and cough without introducing recall bias
- Confounders (sex, childhood SES, respiratory difficulties at birth, childhood phlegm and cough) and adult smoking were accounted to identify significant associations between exposures at age 10 (childhood smoking, parental smoking, and household dampness) and adult phlegm and cough at age 29.
- **The outcome variable that is patterns of two respiratory symptoms (phlegm and cough) was derived extracted from a well-established questionnaire and not indicative of a particular respiratory disease or lung function**.

For peer review only

Abstract

Objective: To examine independent associations between childhood exposures to smoking and housing dampness and **phlegm and cough in adulthood**.

Design: A prospective cohort study.

Participants: 7,320 of the British cohort who were born during one week in 1970 and had complete data for childhood and adult information.

Main outcome measures: Experiences of phlegm and coughing over the previous three months were assessed using questions from the MRC **Questionnaire on respiratory symptoms** when the cohort participants were 29 years of age. Four response patterns (no symptoms, phlegm only, cough only, both symptoms present) were created based on the responses to these questions.

Results: Childhood smoking and exposure to **marked** household dampness at age 10 were associated with **phlegm (childhood smoking: RRR= 1.45, 95%CI=1.02-2.05; dampness: RRR=2.05, 95%CI=1.07-3.91) and co-occurring cough and phlegm (childhood smoking: RRR=1.35. 95%CI=1.08-1.67; dampness: RRR= 2.73, 95%CI=1.88-3.99),** while exposure to **two or more** adult smokers in the household was associated with cough-related symptoms **(cough only: RRR=1.28, 95%CI=1.04-1.58; phlegm and cough: RRR=1.32, 95%CI=1.06-1.64).** These associations were independent from adult smoking, childhood **phlegm and cough**, early social background, and sex. Current smoking at age 29 **contributed to all symptom patters; however, a substantial association between household dampness and co-occurring phlegm and cough suggest long term detrimental effects of childhood environmental exposures.**

Conclusions: Our findings give support to current public health interventions for adult smoking and raise concerns about the long-term effects of a damp home environment on the respiratory health of children.

For peer review only

Introduction

Smoking cessation has been supported as the main preventive measure to reduce smoking-related illnesses and mortality[1]. Previous findings have suggested additional risk factors for adult respiratory health. There are longitudinal links between exposure to parental smoking[2-5] or social disadvantage[6-8] during childhood and adult respiratory health, while research evidence to support the association between indoor dampness and respiratory health is mainly cross-sectional[9-14].

Examining the life course respiratory health of the birth cohort of Britons born in 1946, Mann, Wadsworth and Colley[7] reported that the greatest contribution to adult respiratory health was from proximal risk factors such as adult smoking rather than from distant risk factors such as child respiratory health or social disadvantage. Since adult respiratory health is **likely to be** determined by child respiratory health[15], additional longitudinal effects of childhood exposures, such as smoking and exposure to household damp on adult respiratory health may be small.

However, not many studies have used repeated measures **of** respiratory health **in both childhood and adulthood**. The contribution of childhood exposures to adult respiratory health can be better estimated if a similar respiratory measure is used to assess both child and adult respiratory health. Here we use respiratory symptoms of phlegm and cough occurring in childhood and in adulthood to examine the degree of contribution to **these** adult respiratory **symptoms** from exposure to smoking and household damp during childhood. We used a British cohort who were born in 1970 and examined **this** hypothesis.

1. Household dampness, exposure to adult cigarette smoke during childhood, and childhood smoking will have long-term associations with adult respiratory **symptoms** over and above childhood respiratory **symptoms** and smoking in adulthood.

For peer review only

Method:

Study design, setting and participants

We used information obtained from a prospective cohort study, the 1970 British Cohort Study (BCS70). Detailed information about research design and collected data is given by Elliot and Shepherd[16]. BCS70 targets British residents who were born during one week in 1970; data have been collected regularly across their life course. We used datasets collected at birth[17], at age 10[18] and at age 29[19] to extract study variables. In this study, 7,320 available cases which contain all of the study information were used for the analyses.

Ethical approvals of the study

Data collection at birth and at age 10 was reviewed internally, while ethical approval from the London Multi-centre Research Ethics Committee was obtained for the age 29 survey[20].

Explanatory factors

We used three childhood exposures (child's own smoking, number of adult smokers in the household, household dampness) as explanatory factors. Information about these factors was collected when the cohort children were 10 years of age. A cohort child's own smoking status was indicated by the response to the question: 'Have you tried a cigarette?' Children who had tried a cigarette were coded as 1 as opposed to those who had not yet experimented with smoking (=0). The number of adult smokers in the household was derived through the response about the smoking status of the parents and other adults in the household obtained through a parental interview. This response was tabulated into three categories: 0 for no adult smokers, 1 for one adult, and 2 for two or more adults. **In our study a smoking child could have been exposed to adult smokers at home; however our interest is to assess the role of each factor separately, thus we treated these factors independently.**

In addition, the degree of household dampness was addressed via this parental interview, asking them to rate **the present state of** household dampness as: none, slight, moderate, or

For peer review only

marked. Only a few people responded that their house was 'moderately' damp; therefore, this response was included in the category of 'slight' dampness.

Adulthood factor

Information about adult smoking was collected when the cohort participants were 29 years of age. Their smoking status was indicated as 0 for current non-smokers and 1 for current smokers.

Outcome variable

A pattern of **co-occurring** phlegm and cough **(no symptoms, phlegm only, cough only, phlegm and cough) in adulthood** was the outcome of this study. Cohort participants were asked to respond whether they **usually** brought up phlegm or experienced **coughing** in the morning, during the day or at night in the winter when they were 29 years old. These questions were from the Medical Research Council Questionnaire **on respiratory symptoms** and have been widely used, including in the previous cohort study (i.e. National Child Developmental Study). [21] We adapted the method used by Strachan et al.[22] and created a variable to indicate presence of each respiratory symptom (=phlegm or cough), which cohort participants might have experienced at any time of day. After that we derived a variable with four response patterns of adult respiratory symptoms by combining the response to each variable **obtained at age 29**: 0 no respiratory symptoms, 1 phlegm only, 2 cough only, and 3 both phlegm and cough.

Confounders

We controlled for childhood **phlegm and cough**, sex, paternal social position. **Presence of** phlegm (0=no, 1=yes) or cough (0=no, 1=yes) when the cohort children were 10 years old **was used**. These variables were created through the responses (no, yes but for less than 3 months a year, yes for 3 months or more a year, not known) to the questions: 'Does the study child usually cough/bring up phlegm first thing in the morning/during the day or at night?'

For peer review only

These questions are similar to ones from the MRC Questionnaire, with the omission of the phrase ‘in the winter’. A positive response to a particular respiratory question was treated as presence of the particular respiratory symptom (phlegm or cough), while the response ‘no’ was treated as absence of that respiratory symptom. The response ‘not known’ was regarded as missing and excluded from the analyses. We also included the presence of respiratory difficulties, including respiratory distress syndrome, at the birth of the cohort children (0=absent, 1=present) in the model.

Parental social position was indicated by paternal occupational position at the birth of the cohort child. We adapted the approach by Power et al.[23], classifying the Registrar General’s Social Classes of the cohort children’s father as: 1 – professional or managerial (I& II); 2 – skilled non-manual (III non-manual); 3 – skilled manual (III manual); 4 – partly skilled or unskilled (IV&V) and children who were born in a mother-only household. Midwives provided the information relevant to the birth of the cohort children, while the presence of phlegm or cough was assessed via the parental interview.

Statistical analysis

Multinomial logistic regression was used to identify associations between explanatory factors and patterns of adult respiratory symptoms (phlegm, cough, or both). We took those who did not have any respiratory symptoms as a reference category. **First we entered each explanatory variable in separate models, adjusting for sex. In a final model estimates for all exposures are estimated after adjustment for each other and the confounders.** In our study men and women were analysed together, controlling for the effect of sex. All statistical analyses were performed using Stata SE v12[24].

Results

For peer review only

Study characteristics

Approximately 2.5% of mothers of the cohort children reported that their children brought up phlegm at age 10, while 11% **reported** cough (N=7320). **C**o-occurring symptoms of phlegm and cough **was reported for 8.7% of participants** at age 29, while 2.9% **reported** phlegm only and 9.2% cough **only**. Assessing respiratory symptoms across the life course, **only a** small proportion of participants continuously experienced respiratory symptoms at both times (Table 1).

According to maternal report**s**, less than half of the cohort children grew up in a smoke-free domestic environment, while household dampness of any degree was observed in around 17% of the **homes** of the cohort children. Many of the cohort participants did not smoke at 10 years of age, but around half of the cohort participants were current smokers at age 29. Demographically 48% of the participants were men and 30% of the participants had fathers with non-manual occupations. **Most** of the cohort participants did not experience respiratory difficulties when they were born.

Long-term associations between childhood factors and adult ***phlegm or cough***

The results of multinomial logistic regression showed childhood factors were independently and specifically associated with **patterns of** adult respiratory **symptoms (phlegm or cough)** (Table 2). The relative risk ratio (RRR) for childhood smoking was 1.45 (95%CI=1.02-2.05) for phlegm only and was 1.35 (95%CI=1.08-1.67) for co-occurring phlegm and cough. Growing up in a markedly damp house showed a similar, but stronger association with phlegm only (RRR=2.05, 95%CI=1.07-3.91) and co-occurring phlegm and cough (RRR=2.73, 95%CI=1.88-3.99).

Having adult smokers in the house when the cohort child was 10 years old was positively associated- with all patterns of respiratory symptoms; however, having two or more adult

smokers were significantly associated with adult cohort participants' respiratory symptoms that were cough only (RRR=1.28, 95%CI=1.04-1.58) or co-occurrence of phlegm and cough (RRR=1.32, 95%CI=1.06-1.64). These associations **were** independent of adult smoking and confounders. Adult smoking at age 29 was significantly associated with all of the respiratory response patterns.

For peer review only

Discussion

Key results

Using a British cohort born in 1970, we showed significant longitudinal associations between childhood exposures (child's own smoking, two or more adult smokers in the house, and marked household dampness) and patterns of adult **phlegm or cough**. These were independent of **presence of phlegm or cough at age 10**, adult smoking status, sex and social position of origin. Although the contribution from childhood exposures to adult respiratory **symptom patterns (phlegm, cough or both**) was relatively small compared to adult smoking, household dampness at age 10 showed a substantial contribution to co-occurring phlegm and cough at age 29.

Strengths and limitations

This study benefits from a substantially large sample size with a prospective design which limits recall bias. Questions from the MRC Questionnaire were used to identify the patterns of respiratory symptoms (either phlegm or cough, or both symptoms present) at age 29, rather than the presence of respiratory diseases. We consider that identifying respiratory symptoms of phlegm or cough at age 10, using similar questions to the adult questionnaire, enabled us to estimate associations between childhood exposures and adult respiratory symptom **patterns (phlegm, cough or both)** with considerable accuracy. The omission of the words 'in the

winter' in the child version of the respiratory questions, however, may have over-identified childhood cases of phlegm or cough. In addition, our findings relating to childhood smoking status should be interpreted carefully as they were obtained via self-report. An objective measure of lung function is not available in any sweep of the BCS70 data which is also a limitation to our study.

Information about the duration of exposure to indoor dampness is not available**. In their reports published in 2005, the World Health Organisation[25] recommends detailed and objective assessment of indoor dampness, such as using a trained assessor or measurements of levels of humidity**. In our study we used parental report on household dampness when the cohort children were 10 year old of age (i.e. 1980). This was the only available measure of household dampness. **However, finding a longitudinal association between marked dampness and phlegm related adult respiratory symptoms suggests** that exposure to severe indoor dampness may have irritated the respiratory system of the cohort children, even though they were asymptomatic at that time. **We are unable to account for air pollution, which could affect indoor air quality via ventilation[25] because the information is not available. Further studies with detailed assessments of household dampness and air quality are needed.**

Possible explanations and comparison with previous studies

We found that experimenting with smoking and exposures to marked household dampness at 10 years of age were longitudinally associated with phlegm-related symptoms at age 29, while having two or more adult smokers in the household showed detrimental associations with cough-related symptoms at the same age. These longitudinal associations are independent of previous **phlegm or cough**, adult smoking, and other confounders. It is worth noting that nearly 80% of the cohort participants (n=11,648) did not have any respiratory

symptoms at age 10 to start with, yet 20% of these participants had developed respiratory symptoms, phlegm or cough by age 29. This is somewhat similar to the findings by Guerra et al.[3] that lung function became markedly poorer at age 26 only when participants were exposed to both parental smoking at their birth and were **smokers themselves**. It is possible that **development of phlegm or cough** may not coincide with earlier exposures to smoking or household dampness. Our findings also add to the existing findings **that** link parental smoking and respiratory symptoms in adulthood [2 4] and indicate that the mode (active vs. second-hand) of childhood exposure to smoking may affect respiratory systems differently. Our finding linking household dampness with presence of phlegm in adulthood is similar to the findings by Sahlberg et al.[26] that adults exposed to household dampness showed increased occurrence of mucosal symptoms 10 years **later**. **Our finding is also similar to the meta analyses results by Mendell et al.[27], showing a substantial association between indoor dampness and various respiratory symptoms, including cough.**

Our finding supports longitudinal associations between childhood exposures and adult respiratory **symptoms** similarly to the study by Mann et al.[7] **We are** the first to show a substantial contribution of childhood exposure to household dampness to co-occurring phlegm and cough at age 29.

Implications and conclusions

In summary, we **found** support **for** longitudinal associations between childhood exposures to smoking and household dampness, and **patterns of** adult respiratory **symptoms**. Although **adult smoking contributed to all of** adult respiratory **symptom patterns,** exposure to **marked** household dampness during childhood showed a substantial contribution to co-occurring phlegm and cough. Our findings support current public health interventions to reduce adult smoking, but also indicate that the management of childhood risk factors such as

For peer review only

exposure to smoke (active or second-hand) and household dampness can be a way to prevent adults experiencing poor respiratory health.

For peer review only

Footnotes:

Contributors: NC planned the study, analysed the data, and participated in writing and discussion. AS advised statistical analyses and participated in writing and discussion. MB, YK and YS participated in writing and discussion.

Funding information: This work has funded through the Economic and Social Research Council's International Centre for Life Course Studies in Society and Health (ES/J019119/1).

Conflicts of Interests: None declared

Ethical approval: BCS70 data used in this study were managed by the Centre for Longitudinal Study and accessible via the UK data archive. The data do not contain identifiable information and academic use of the BCS70 data does not require ethical approvals.

Data sharing statement: BCS70 data used in this study have been managed by the Centre for Longitudinal Study and are accessible via the UK data archive. The statistical code for derived variables is available from the correspondence author.

For peer review only

Table 1. Description (%) of explanatory and confounding variables by respiratory conditions (no symptoms, phlegm only, cough only, phlegm and cough) at age 29 (N=7,320)

	No symptoms % (n=5792)	Phlegm % (n=214)	Cough % (n=675)	Phlegm + Cough % (n=639)
**Explanatory variables**				
Smoking status at age 10				
Never	88.09	78.97	84.74	79.50
Smoked	11.92	21.12	15.26	20.50
Household dampness at age 10				
No dampness	84.34	83.18	84.15	76.37
Slight to moderate	13.33	11.68	12.40	16.91
Marked	2.33	5.14	3.41	6.73
Numbers of adult smokers in the household at age 10				
Zero	36.08	28.50	29.93	26.60
One	36.31	38.32	35.83	38.50
Two or more	27.61	33.18	34.22	34.90
Smoking status at age 29				
Current smoker	49.64	70.56	70.81	80.44
**Confounders**				
Phlegm at age 10				
present	2.05	5.61	4.00	4.38
Cough at age 10				
Present	9.94	14.49	14.37	16.28
Respiratory difficulties at birth				
Present	2.14	2.34	2.22	2.19
Social position of origin				
**Professional & managerial**	18.42	20.56	14.67	13.77
**Skilled non-manual**	14.50	11.21	13.04	10.33
**Skilled manual**	45.51	42.99	46.96	48.36
**Non-skilled manual + no male head**	21.56	25.23	25.33	27.54
Sex				
Male	45.98	67.76	45.63	63.85
Female	54.02	32.24	54.37	36.15

For peer review only

Table 2. Associations between child and adult risk factors and adult respiratory conditions expressed as relative risk ratios (RRR) and 95% **confidence intervals in parentheses** obtained by multinomial logistic regression (N=7320)

	No symptoms (n=5792)	Phlegm only (n=214)		Cough only (n=675)		Phlegm + Cough (n=639)	
		Bivariate[a]	Fully adjusted[b]	Bivariate[a]	Fully adjusted[b]	Bivariate[a]	Fully adjusted[b]
**Explanatory factors**							
Smoking status at age 10 (Reference: Not smoked)	Ref	**1.74 (1.24-2.45)**	1.45 (1.02-2.05)	**1.34 (1.07-1.68)**	1.12 (0.89-1.41)	**1.72 (1.40-2.13)**	1.35 (1.08-1.67)
Household dampness at age 10 (Reference: no dampness)							
Slight to moderate	Ref	**0.89 (0.58-1.37)**	0.82 (0.54-1.27)	**0.94 (0.74-1.19)**	0.85 (0.67-1.09)	**1.40 (1.12-1.75)**	1.24 (0.99-1.56)
Marked	Ref	**2.33 (1.23-4.41)**	2.05 (1.07-3.91)	**1.46 (0.93-2.30)**	1.26 (0.80-1.99)	**3.30 (2.31-4.73)**	2.73 (1.88-3.99)
Numbers of adult smokers in the household at age 10 (reference: Zero)							
One	Ref	**1.35 (0.96-1.90)**	1.23 (0.88-1.74)	**1.19 (0.98-1.45)**	1.07 (0.87-1.30)	**1.45 (1.18-1.78)**	1.20 (0.97-1.49)
Two or more	Ref	**1.54 (1.09-2.19)**	1.36 (0.95-1.95)	**1.49 (1.22-1.82)**	1.28 (1.04-1.58)	**1.74 (1.41-2.15)**	1.32 (1.06-1.64)
Current smoker at age 29 (Reference: Non-smoking)	Ref	**2.40 (1.78-3.24)**	2.29 (1.69-3.09)	**2.46 (2.07-2.93)**	2.41 (2.02-2.88)	**4.13 (3.37-5.06)**	3.95 (3.22-4.85)
**Confounders**							
Phlegm present at age 10 (Reference: Absent)	Ref	--	2.62 (1.28-5.39)	--	1.60 (0.99-2.58)	--	1.63 (1.01-2.64)
Cough present at age 10 (Reference: Absent)	Ref	--	1.14 (0.72-1.81)	--	1.34 (1.03-1.74)	--	1.45 (1.12-1.89)
Sex (Reference: Men)	Ref	--	0.42 (0.31-0.56)	--	1.03 (0.87-1.21)	--	0.49 (0.41-0.59)
Respiratory difficulties at birth	Ref	--	1.00	--	1.06	--	0.97

For peer review only

For peer review only - http://bmjopen.bmj.com/site/about/guidelines.xhtml

(Reference: Absence)			(0.40-2.49)		(0.62-1.84)		(0.54-1.72)
Social position of origin (Reference: **Professional and managerial**)							
**Skilled non-manual**	Ref	--	0.68 (0.41-1.13)	--	1.14 (0.84-1.54)	--	0.95 (0.68-1.33)
**Skilled manual**	Ref	--	0.81 (0.56-1.18)	--	1.27 (1.00-1.62)	--	1.36 (1.05-1.76)
**Non-skilled manual** + no male head	Ref	--	0.94 (0.61-1.42)	--	1.37 (1.05-1.79)	--	1.46 (1.10-1.93)

[a] **Estimates are adjusted for sex**

[b] **Estimates for explanatory factors are adjusted for sex, confounders and other exposures**

For peer review only

References

1. Department of Health. Healthy lives, healthy people: Our strategy for public health in England. London: Department of Health, 2010:1-95.

2. Gray LA, Leyland AH, Benzeval M, Watt GC. Explaining the social patterning of lung function in adulthood at different ages: the roles of childhood precursors, health behaviours and environmental factors. *J Epidemiol Community Health* 2013;67(11):905-11.

3. Guerra S, Stern DA, Zhou M, Sherrill DL, Wright AL, Morgan WJ, et al. Combined effects of parental and active smoking on early lung function deficits: a prospective study from birth to age 26 years. *Thorax* 2013.

4. Svanes C, Omenaas E, Jarvis D, Chinn S, Gulsvik A, Burney P. Parental smoking in childhood and adult obstructive lung disease: results from the European Community Respiratory Health Survey. *Thorax* 2004;59(4):295-302.

5. Beyer D, Mitfessel H, Gillissen A. Maternal smoking promotes chronic obstructive lung disease in the offspring as adults. *Eur. J. Med. Res.* 2009;14 Suppl 4:27-31.

6. Bartley M, Kelly Y, Sacker A. Early life financial adversity and respiratory function in midlife: a prospective birth cohort study. *Am J Epidemiol* 2012;175(1):33-42.

7. Mann SL, Wadsworth ME, Colley JR. Accumulation of factors influencing respiratory illness in members of a national birth cohort and their offspring. *J Epidemiol Community Health* 1992;46(3):286-92.

8. Jackson B, Kubzansky LD, Cohen S, Weiss S, Wright RJ. A matter of life and breath: childhood socioeconomic status is related to young adult pulmonary function in the CARDIA study. *Int J Epidemiol* 2004;33(2):271-8.

9. Kilpelainen M, Terho EO, Helenius H, Koskenvuo M. Home dampness, current allergic diseases, and respiratory infections among young adults. *Thorax* 2001;56(6):462-7.

For peer review only

10. Norback D, Zock JP, Plana E, Heinrich J, Svanes C, Sunyer J, et al. Mould and dampness in dwelling places, and onset of asthma: the population-based cohort ECRHS. *Occup Environ Med* 2013;70(5):325-31.

11. Pirastu R, Bellu C, Greco P, Pelosi U, Pistelli R, Accetta G, et al. Indoor exposure to environmental tobacco smoke and dampness: respiratory symptoms in Sardinian children--DRIAS study. *Environ. Res.* 2009;109(1):59-65.

12. Simoni M, Lombardi E, Berti G, Rusconi F, La Grutta S, Piffer S, et al. Mould/dampness exposure at home is associated with respiratory disorders in Italian children and adolescents: the SIDRIA-2 Study. *Occup Environ Med* 2005;62(9):616-22.

13. Zock JP, Jarvis D, Luczynska C, Sunyer J, Burney P. Housing characteristics, reported mold exposure, and asthma in the European Community Respiratory Health Survey. *J. Allergy Clin. Immunol.* 2002;110(2):285-92.

14. Weinmayr G, Gehring U, Genuneit J, Buchele G, Kleiner A, Siebers R, et al. Dampness and moulds in relation to respiratory and allergic symptoms in children: results from Phase Two of the International Study of Asthma and Allergies in Childhood (ISAAC Phase Two). *Clin. Exp. Allergy* 2013;43(7):762-74.

15. Tennant PW, Gibson GJ, Parker L, Pearce MS. Childhood respiratory illness and lung function at ages 14 and 50 years: childhood respiratory illness and lung function. *Chest* 2010;137(1):146-55.

16. Elliott J, Shepherd P. Cohort Profile: 1970 British Birth Cohort (BCS70). *International Journal of Epidemiology* 2006;35 836-43.

17. Chamberlain R, Chamberlain G, University of London Institute of Education Centre for Longitudinal Studies. 1970 Birth Cohort Study: Birth and 22-month Subsample, 1970-1972. 3rd edition ed: UK Data Archive University of Essex Colchester, 2013.

For peer review only

18. Butler N, Bynner JM, University of London Institute of Education Centre for Longitudinal Studies. 1970 British Birth Cohort Study: Ten-Year Follow-up, 1980. 4th edition ed. Colchester: UK data Archive University of Essex Colchester, 2013.

19. University of London Institute of Education Centre for Longitudinal Studies. 1970 British Birth Cohort Study: Twenty-Nine-Year Follow-up, 1999-2000. 3rd edition ed. Colchester: UK Data Archive University of Essex Colchester, 2013.

20. Shepherd P. 1979 British Cohort Study Ethical Review and consent. London: Centre for Longitudinal Studies, 2012:1-14.

21. Power C, Elliott J. Cohort profile: 1958 British birth cohort (National Child Development Study). *International Journal of Epidemiology* 2006;35(1):34-41.

22. Strachan DP, Anderson HR, Bland JM, Peckham C. Asthma as a link between chest illness in childhood and chronic cough and phlegm in young adults. *Br. Med. J. (Clin. Res. Ed).* 1988;296(6626):890-3.

23. Howe LD, Hargreaves JR, Ploubidis GB, De Stavola BL, Huttly SR. Subjective measures of socio-economic position and the wealth index: a comparative analysis. *Health Policy Plan.* 2011;26(3):223-32.

24. Stata Statistical Software: Release 12 [program]. V12.0 version. College Station, TX: StataCorp LP, 2011.

25. World Health Organization. WHO guidelines for indoodr air quality: dampness and mould. In: Heseltine E, Rosen J, editors. Copenhagen, 2009:1-248.

26. Sahlberg B, Norback D, Wieslander G, Gislason T, Janson C. Onset of mucosal, dermal, and general symptoms in relation to biomarkers and exposures in the dwelling: a cohort study from 1992 to 2002. *Indoor Air* 2012;22(4):331-8.

For peer review only

27. Mendell MJ, Mirer AG, Cheung K, Tong M, Douwes J. Respiratory and Allergic Health Effects of Dampness, Mold, and Dampness-Related Agents: A Review of the Epidemiologic Evidence. *Environ. Health Perspect.* 2010;119:748-56.

For peer review only

STROBE Statement—checklist of items that should be included in reports of observational studies

	Item No	Recommendation
**Title and abstract**	1	(*a*) Indicate the study's design with a commonly used term in the title or the abstract
		(*b*) Provide in the abstract an informative and balanced summary of what was done and what was found
**Introduction**		
Background/rationale	2	Explain the scientific background and rationale for the investigation being reported
Objectives	3	State specific objectives, including any prespecified hypotheses
**Methods**		
Study design	4	Present key elements of study design early in the paper
Setting	5	Describe the setting, locations, and relevant dates, including periods of recruitment, exposure, follow-up, and data collection
Participants	6	(*a*) *Cohort study*—Give the eligibility criteria, and the sources and methods of selection of participants. Describe methods of follow-up<br>*Case-control study*—Give the eligibility criteria, and the sources and methods of case ascertainment and control selection. Give the rationale for the choice of cases and controls<br>*Cross-sectional study*—Give the eligibility criteria, and the sources and methods of selection of participants
		(*b*) *Cohort study*—For matched studies, give matching criteria and number of exposed and unexposed<br>*Case-control study*—For matched studies, give matching criteria and the number of controls per case
Variables	7	Clearly define all outcomes, exposures, predictors, potential confounders, and effect modifiers. Give diagnostic criteria, if applicable
Data sources/ measurement	8*	For each variable of interest, give sources of data and details of methods of assessment (measurement). Describe comparability of assessment methods if there is more than one group
Bias	9	Describe any efforts to address potential sources of bias
Study size	10	Explain how the study size was arrived at
Quantitative variables	11	Explain how quantitative variables were handled in the analyses. If applicable, describe which groupings were chosen and why
Statistical methods	12	(*a*) Describe all statistical methods, including those used to control for confounding
		(*b*) Describe any methods used to examine subgroups and interactions
		(*c*) Explain how missing data were addressed
		(*d*) *Cohort study*—If applicable, explain how loss to follow-up was addressed<br>*Case-control study*—If applicable, explain how matching of cases and controls was addressed<br>*Cross-sectional study*—If applicable, describe analytical methods taking account of sampling strategy
		(*e*) Describe any sensitivity analyses

Continued on next page

For peer review only - http://bmjopen.bmj.com/site/about/guidelines.xhtml

**Results**		
Participants	13*	(a) Report numbers of individuals at each stage of study—eg numbers potentially eligible, examined for eligibility, confirmed eligible, included in the study, completing follow-up, and analysed
		(b) Give reasons for non-participation at each stage
		(c) Consider use of a flow diagram
Descriptive data	14*	(a) Give characteristics of study participants (eg demographic, clinical, social) and information on exposures and potential confounders
		(b) Indicate number of participants with missing data for each variable of interest
		(c) *Cohort study*—Summarise follow-up time (eg, average and total amount)
Outcome data	15*	*Cohort study*—Report numbers of outcome events or summary measures over time
		Case-control study—Report numbers in each exposure category, or summary measures of exposure
		Cross-sectional study—Report numbers of outcome events or summary measures
Main results	16	(*a*) Give unadjusted estimates and, if applicable, confounder-adjusted estimates and their precision (eg, 95% confidence interval). Make clear which confounders were adjusted for and why they were included
		(*b*) Report category boundaries when continuous variables were categorized
		(*c*) If relevant, consider translating estimates of relative risk into absolute risk for a meaningful time period
Other analyses	17	Report other analyses done—eg analyses of subgroups and interactions, and sensitivity analyses
**Discussion**		
Key results	18	Summarise key results with reference to study objectives
Limitations	19	Discuss limitations of the study, taking into account sources of potential bias or imprecision. Discuss both direction and magnitude of any potential bias
Interpretation	20	Give a cautious overall interpretation of results considering objectives, limitations, multiplicity of analyses, results from similar studies, and other relevant evidence
Generalisability	21	Discuss the generalisability (external validity) of the study results
**Other information**		
Funding	22	Give the source of funding and the role of the funders for the present study and, if applicable, for the original study on which the present article is based

*Give information separately for cases and controls in case-control studies and, if applicable, for exposed and unexposed groups in cohort and cross-sectional studies.

Note: An Explanation and Elaboration article discusses each checklist item and gives methodological background and published examples of transparent reporting. The STROBE checklist is best used in conjunction with this article (freely available on the Web sites of PLoS Medicine at http://www.plosmedicine.org/, Annals of Internal Medicine at http://www.annals.org/, and Epidemiology at http://www.epidem.com/). Information on the STROBE Initiative is available at www.strobe-statement.org.

For peer review only
